# Increase in Social Isolation during the COVID-19 Pandemic and Its Association with Mental Health: Findings from the JACSIS 2020 Study

**DOI:** 10.3390/ijerph18168238

**Published:** 2021-08-04

**Authors:** Hiroshi Murayama, Ryo Okubo, Takahiro Tabuchi

**Affiliations:** 1 Research Team for Social Participation and Community Health, Tokyo Metropolitan Institute of Gerontology, Tokyo 173-0015, Japan; 2 Department of Clinical Epidemiology, Translational Medical Center, National Center of Neurology and Psychiatry, Tokyo 187-8551, Japan; ryo-okubo@ncnp.go.jp; 3 Cancer Control Center, Osaka International Cancer Institute, Osaka 541-8567, Japan; tabuti-ta@mc.pref.osaka.jp

**Keywords:** COVID-19, mental health, Japan, prevalence, social isolation

## Abstract

The coronavirus disease 2019 (COVID-19) pandemic is assumed to have caused an increase in the number of socially isolated people. However, the prevalence of social isolation during the pandemic has not been well studied, particularly among Asian populations. This study investigated changes in the prevalence of social isolation caused by the COVID-19 pandemic and examined its association with mental health among the general Japanese population. Data were obtained from a large-scale, web-based nationwide survey conducted from August to September 2020 (*n* = 28,000; aged 15–79 years). Social isolation was defined as less frequent contact with people other than co-residing family members. We assessed the participants’ frequency of contact in January (before the pandemic) and August 2020 (during the pandemic). Mental health outcomes included psychological distress, suicidal ideation, loneliness, and fear of COVID-19. We analyzed the data of 25,482 respondents. The weighted prevalence (95% confidence interval) of social isolation was 21.2% (20.7–21.7%) and 27.9% (27.3–28.4%) before and during the pandemic, respectively. The prevalence of social isolation increased by 6.7 (6.3–7.0) percentage points during the pandemic. Older people and men had the greatest increase in the prevalence of social isolation. People who became socially isolated during the pandemic had greater loneliness and fear of COVID-19 than those who were consistently not socially isolated since before the pandemic. This study suggested that social isolation had increased during the COVID-19 pandemic in Japan. Our findings highlight the importance of developing immediate measures against social isolation to maintain good mental health.

## 1. Introduction

Social isolation has deleterious effects on health outcomes, such as all-cause mortality [1,2], coronary heart disease [3], hypertension [4], depression [5,6], dementia [7], suicidal ideation [8,9,10], smoking behaviors [11], and well-being risk [12]. Social isolation may be an important factor for adverse health outcomes.

The coronavirus disease 2019 (COVID-19) pandemic is assumed to have caused an increase in the number of socially isolated people. For example, social distancing, which has been recommended by the World Health Organization and the Centers for Disease Control and Prevention to reduce the risk of infection, has possibly increased social isolation [13]. Indeed, people who maintained more social distancing were likely to have poor physical and mental health status, which may have been caused by social isolation [14,15]. Moreover, during the COVID-19 pandemic, it has been reported that socially isolated older adults had difficulty seeking practical help [16]. Therefore, public health researchers and policymakers have been concerned about the deleterious effects of social isolation on individuals’ health during the pandemic.

Despite these backgrounds, there is limited research on the prevalence of social isolation during the pandemic. Peng and Roth examined changes in social isolation using data from the Health and Retirement Study and found an increase in the prevalence of social isolation among people aged ≥ 50 years during the COVID-19 social distancing policy implementation period in the United States [17]. However, their sample only included middle- and old-aged people. In addition, this study was conducted in a Western country. As the pandemic situation, as well as socio-cultural and institutional backgrounds, in non-Western countries, including Asian countries, differ from those in Western countries, a study should be conducted in each region. Moreover, the prevalence and consequences of social isolation during the pandemic have not been fully investigated.

This study used data from a large-scale, web-based nationwide survey conducted during the COVID-19 pandemic among Japanese people and investigated changes in the prevalence of social isolation caused by the pandemic. We also examined the association between social isolation and mental health outcomes. This information may contribute to the development of effective measures for social isolation during and after the pandemic.

## 2. Materials and Methods

### 2.1. Study Design and Participants

Data were obtained from a baseline survey of the Japan “COVID-19 and Society” Internet Survey (JACSIS) study, conducted in 2020. The JACSIS study was a national representative, web-based, self-reported questionnaire survey that used a large internet survey agency (Rakuten Insight Inc., Tokyo, Japan; https://in.m.aipsurveys.com; accessed on 1 August 2021). This agency had approximately 2.2 million registered panelists in 2019.

In the survey, 224,389 panelists (men and women aged 15–79 years) were invited using random sampling stratified by sex, age, and prefecture (covering all 47 prefectures). The questionnaire was distributed to panelists from 25 August to 30 September 2020. We determined a sample size of 28,000 panelists based on the population distribution in 2019. Therefore, the survey was terminated once the target number of respondents was reached for each category (sex, age, and prefecture). Consequently, 28,000 panelists responded to the survey. Supplementary Materials Figure S1 presents the number of new COVID-19 cases reported each day in Japan until November 2020. The survey was conducted in the latter half of the second wave of the pandemic in Japan.

This study was reviewed and approved by the Research Ethics Committee of Osaka International Cancer Institute (approved on 19 June 2020; approval number: 20084). Web-based informed consent was obtained from all participants before they responded to the questionnaire, and the option to opt-out at any point was provided. The internet survey agency respected the Act on the Protection of Personal Information in Japan. A credit point system known as “E-points”, which could be used for internet shopping and cash conversion, was offered as an incentive.

To validate the quality of the data, we excluded responses with discrepancies and/or artificial/unnatural responses. The following criteria were used: (1) An invalid response to “Please choose the second alternative from the bottom” (i.e., panelists who failed to select the second from last alternative from the five options available); (2) positive responses to all questions related to drug use (e.g., marijuana, cocaine or heroin); and (3) positive responses to all questions regarding underlying 16 alternative chronic diseases. We excluded 2518 respondents with discrepancies and/or artificial/unnatural responses (remaining respondents, *n* = 25,482).

### 2.2. Measures

#### 2.2.1. Social Isolation

Previous studies defined social isolation as less frequent contact with people other than co-residing family members [18,19,20]. These studies captured each participant’s frequency of direct and indirect contact. In this study, we assessed the frequency of contact using the following eight questions at two time-points (January and August 2020): (1) “How often did you see your family members or relatives who are living apart?”; (2) “How often did you make contact with your family members or relatives who are living apart by email or text message (e.g., mobile, LINE, Facebook Messenger)?”; (3) “How often did you make contact with your family members or relatives who are living apart by voice call (e.g., telephone, mobile, LINE, Facebook Messenger, Skype)?”; (4) “How often did you make contact with your family members or relatives who are living apart by video call (e.g., LINE, Facebook Messenger, Skype, Zoom)?”; (5) “How often did you see your friends or neighbors?”; (6) “How often did you make contact with your friends or neighbors by email or text message?”; (7) “How often did you make contact with your friends or neighbors by voice call?”; and (8) “How often did you make contact with your friends or neighbors by video call?” The participants answered using the following seven response options: “almost every day (6–7 times a week)”, “4–5 times a week”, “2–3 times a week”, “once a week”, “2–3 times a month”, “once a month”, and “rarely”.

We calculated the overall frequency of contact in January and August 2020, using the following method based on a previous study on social isolation [18]. First, we assumed the frequency of contact per month based on the response category. The average number of weeks in a month is 4.35 (=365 [days in a year]/12 [months in a year]/7 [days in a week]). Therefore, for example, if a respondent selected “almost every day (6–7 times a week)”, we calculated the frequency as 28.28 days per month (=6.5 [days in a week] × 4.35 [weeks in a month]). Using the same method, we calculated “4–5 times a week”, “2–3 times a week”, “once a week”, “2–3 times a month”, “once a month”, and “rarely” as 19.58 days (=4.5 [days in a week] × 4.35 [weeks in a month]), 10.88 days (=2.5 [days in a week] × 4.35 [weeks in a month]), 4.35 days (=1 [day in a week] × 4.35 [weeks in a month]), 2.5 days, 1 day, and 0 days, respectively. Second, we summarized the frequency of contact (days in a month) based on the abovementioned eight questions. If a respondent answered “2–3 times a month” for all eight questions, we calculated the frequency as 20 days per month (=2.5 [days in a month] × 8 [questions]). Third, based on the overall frequency of contact, we considered those who made contacts less than once per week (i.e., 4.35 days per month) as those experiencing social isolation, since a frequency of less than once a week has been shown to be associated with greater risk of all-cause mortality, dementia, and disability [18].

#### 2.2.2. Mental Health

We included psychological distress, suicidal ideation, loneliness, and fear of COVID-19 as mental health outcomes. Psychological distress was assessed using the Kessler Psychological Distress Scale (K6) [21]. The K6 has been widely used in epidemiological studies to measure psychological distress among the general population, and it consists of six questions. Respondents answered each question on a five-point scale (“0 = never”, “1 = rarely”, “2 = sometimes”, “3 = often” or “4 = always”). The score range was 0–24, and a score of ≥13 indicated severe psychological distress [21,22].

Suicidal ideation was assessed by asking one question “Have you ever wished you were dead, since April 2020?” (“1 = experienced it for the first time”, “2 = experienced it before April 2020” or “3 = never experienced it”). To analyze newly emerged suicidal ideation, we excluded 2060 individuals who reported suicidal ideation before April 2020.

The University of California, Los Angeles (UCLA) Loneliness Scale version 3, Short Form 3-item (UCLA-LS3-SF3) was used to assess loneliness [23,24]. The validity and reliability of the Japanese version of the UCLA-LS3-SF3 were confirmed [25]. Participants responded to each item on a five-point scale (“never”, “rarely”, “sometimes”, “often” or “always”). Since the original UCLA-LS3-SF3 used a four-point scale (“never”, “rarely”, “sometimes” or “always”), we combined the “often” and “always” categories after checking the distribution of responses (i.e., “1 = never”, “2 = rarely”, “3 = sometimes” or “4 = often/always”). The score range was 3–12, with higher scores indicating severe loneliness. 

Fear related to COVID-19 was assessed using the Fear of COVID-19 Scale (FCV-19S), which consists of seven items with a five-point response scale [26]. The FCV-19S was validated in Japan [27]. The score range was 7–35, with higher scores indicating greater fear of COVID-19.

#### 2.2.3. Covariates

Age, sex, marital status, household composition, education, annual household income, working conditions, and house ownership were included as sociodemographic characteristics. Health behaviors (smoking status, drinking habit, and frequency of vigorous physical activity) and the presence of chronic diseases (hypertension, angina, myocardial infarction, stroke, diabetes mellitus, chronic obstructive pulmonary disease, and cancer) were also included as covariates.

In addition, environmental factors (urbanization level and prefectural COVID-19 outbreak situation) were considered. As a proxy indicator of urbanization level, we used Densely Inhabited District (DID) data, based on the 2015 Population Census of Japan. Any DID without a zip-code centroid was classified as a non-DID (rural district) and placed in the lowest urbanization level category. Any DID with a zip code centroid was classified as a DID (urban district) and classified into tertiles according to the population density. Regarding the prefectural COVID-19 outbreak situation, participants were stratified into four groups based on quartiles of the cumulative number of confirmed cases per 100,000 population between 15 January 2020 (the day the first case of COVID-19 was confirmed in Japan) and 25 August 2020 (the first day of the survey) in each residential prefecture.

### 2.3. Statistical Analyses

We estimated the prevalence of social isolation in the total sample according to sociodemographic characteristics. In describing the prevalence of social isolation, we used sampling weights to adjust for biased demographic distribution using national vital statistics (as of 1 June 2020). Sampling weights were calculated by age (5-year intervals) and sex by multiplying the selection probabilities by the inverse of the participation rate for all 26 subgroups. The difference in prevalence before and during the pandemic was tested using the chi-square test. The variation of the difference in prevalence according to sociodemographic characteristics and environmental factors was compared using the t-test or one-way analysis of variance. Next, we examined the association between social isolation and mental health outcomes. We used binary logistic regression analyses for psychological distress and suicidal ideation, and multiple linear regression analyses for loneliness and fear of COVID-19, adjusting for sociodemographic characteristics, health behaviors, and the presence of chronic diseases. All of the tests were two-sided with a significance level of <0.05. Statistical analyses were performed using IBM SPSS 23 (IBM Corp., Armonk, NY, USA).

## 3. Results

Table 1 presents the prevalence of social isolation among the participants by age and sex. The weighted prevalence of social isolation in the total sample was 21.2% (95% confidence interval [CI]: 20.7–21.7%) before the pandemic (January 2020). Middle-aged people tended to have a higher prevalence of social isolation than younger and older people (*p* < 0.001). However, during the pandemic (August 2020), the prevalence of social isolation significantly increased by 6.7 (6.3–7.0) percentage points (*p* < 0.001), to 27.9% (27.3–28.4%). The prevalence of social isolation significantly increased across all age and sex strata (*p* < 0.001). In particular, the prevalence of social isolation was higher among men before and during the pandemic (*p* < 0.001), and the increase in the prevalence of social isolation was greater among men than among women (7.6 [7.0–8.2] vs. 5.6 [5.2–6.2] percentage points, respectively; *p* < 0.001). The increase in the prevalence of social isolation was also greater in older people both in men and women (*p* < 0.001). The weighted prevalence of the frequency of contact based on the aforementioned eight items by age and sex are shown in Table S1.

We compared the prevalence of social isolation by sociodemographic characteristics (Table 2). The prevalence of social isolation significantly increased across all sociodemographic strata (*p* < 0.001). There were significant disparities in the prevalence of social isolation by socioeconomic status (SES) before the pandemic. People with lower education and annual household income had a higher prevalence of social isolation (*p* < 0.001). However, the increase in the prevalence of social isolation during the pandemic did not differ significantly by SES (*p* = 0.303 for education; *p* = 0.074 for annual household income), indicating that these socioeconomic disparities remained during the pandemic. On the contrary, the increase in the prevalence of social isolation during the pandemic among those who were married, lived with others, and did not own their house was significantly greater than that among their counterparts (*p* < 0.001, *p* = 0.001, and *p* < 0.001, respectively). Table S2 presents the prevalence of social isolation during the pandemic by sociodemographic characteristics, by sex.

Moreover, in Table 3, we calculated the prevalence of social isolation by urbanization level and prefectural COVID-19 outbreak situation (the cumulative number of confirmed COVID-19 cases per 100,000 population between 15 January and 25 August 2020; median, 20.3 per 100,000). The prevalence of social isolation significantly increased across all strata (*p* < 0.001). However, no significant difference in the prevalence of social isolation before and during the pandemic was detected by urbanization level (*p* = 0.070) or prefectural COVID-19 outbreak situation (*p* = 0.912).

Table 4 presents the prevalence of the transition of social isolation status before and during the COVID-19 pandemic. The proportion of respondents who were not socially isolated before and during the pandemic (i.e., “non-SI to non-SI”; SI, socially isolated) was 70.4% (70.2–70.7%), while that of those who were socially isolated before and during the pandemic (i.e., “SI to SI”) was 19.5% (19.3–19.8%). The proportion of people who became socially isolated during the pandemic (i.e., “non-SI to SI”) was 8.3% (8.2–8.5%), which was higher among men than among women (9.4% [9.2–9.7%] vs. 7.3% [7.0–7.5%]; *p* < 0.001).

Table 5 shows the association between the transition of social isolation status and mental health outcomes. The Cronbach’s alphas for K6, UCLA-LS3-SF3, and FCV-19S in this study were 0.94, 0.93, and 0.87, respectively. There was no association between social isolation status and psychological distress, as assessed by K6, after adjusting for sociodemographic characteristics, health behaviors, and the presence of chronic diseases. Compared to persistently non-socially isolated people (“non-SI to non-SI”), those who were persistently socially isolated (“SI to SI”) tended not to have suicidal ideation (odds ratio, 0.687 [95% CI: 0.561–0.842]), while social isolation during the COVID-19 pandemic (“non-SI to SI”) was not associated with suicidal ideation. In contrast, compared to those who were persistently not socially isolated (“non-SI to non-SI”), those who became socially isolated during the pandemic (“non-SI to SI”) were more likely to be lonely (β [standardized coefficient] = 0.020; *p* = 0.001) and have a greater fear of COVID-19 (β = 0.028; *p* < 0.001), while those who were persistently socially isolated (“SI to SI”) or became non-socially isolated during the pandemic (“SI to non-SI”) were not likely to be lonely or fearful of COVID-19.

## 4. Discussion

This study investigated social isolation status during the COVID-19 pandemic in a large, nationwide population with a wide age range (15–79 years). The prevalence of social isolation increased in all age groups, particularly among men and older age groups. Moreover, social isolation during the COVID-19 pandemic was associated with greater loneliness and fear of COVID-19. To our knowledge, this is the first study to analyze the prevalence of social isolation during the COVID-19 pandemic in an Asian country, providing valuable evidence of the actual situation regarding social isolation during the pandemic.

The prevalence of social isolation increased significantly by 6.7 percentage points during the COVID-19 pandemic. This was consistent with a study of middle-aged and older adults in the United States, which reported an increase in the prevalence of social isolation [17]. We also observed that the increased trend was more marked in men and older people. A Japanese study, conducted in 2003, reported that 15.8% of people aged ≥65 years were socially isolated (defined as having contact less than once a week) and that the prevalence of social isolation was higher in men than in women (21.2% vs. 10.6%, respectively) [18]. The prevalence of social isolation in Japan has increased from 2010 to 2016, although the increase was relatively small for both sexes [28]. These findings suggest that the pandemic (including infection prevention policies, such as social distancing) has had a severe impact on social isolation, particularly in older people. The pandemic has encouraged our use of online communication tools. The social isolation indicator used in this study included contact with others through online communication tools, such as Zoom and Skype. However, older people were not familiar with the internet in Japan [29]. Thus, social isolation increased among older populations, while it tended to be less prevalent among younger populations, during the pandemic. This indicates that the pandemic widened the age disparity in social isolation.

Regarding SES, although there were socioeconomic disparities in the prevalence of social isolation before the pandemic, we observed similar increases in each SES category. This suggests that the pandemic has universally influenced people’s social connections with others, regardless of their socioeconomic background.

We found no association between the prevalence of social isolation during the COVID-19 pandemic and psychological distress and suicidal ideation. Generally, social isolation has adverse effects on people’s health, including mental health [1,2,3,4,5,6,7,8,9,10,11,12]. However, evidence on the association between social isolation during the COVID-19 pandemic and health status has been inconsistent. This may be partly due to the effect of confounding factors on the association between the prevalence of social isolation and psychological distress during the pandemic. Several studies have reported a link between lockdown or social distancing policies and poor mental health [30,31,32,33], including a Japanese study [33]. Since lockdown or social distancing policies may also lead to an increase in the prevalence of social isolation, it is difficult to distinguish the effects of lockdown or social distancing policies from the effects of social isolation on mental health. Social isolation has potential medium- and long-term effects, as well as short-term effects, on mental health, and these effects on mental health should be evaluated.

Conversely, people who became socially isolated during the COVID-19 pandemic experienced greater loneliness than those who were not persistently socially isolated. According to the Evolutionary Theory of Loneliness [34], social isolation and loneliness interact with each other and may reinforce and perpetuate each other over time. However, those who became socially isolated during the COVID-19 pandemic experienced greater loneliness than those who were persistently socially isolated (β = 0.020 for the “non-SI to SI” subgroup and 0.009 for the “SI to SI” subgroup). This implied that social isolation during the COVID-19 pandemic seriously impacted the feeling of loneliness.

Social isolation during the COVID-19 pandemic was associated with greater fear of COVID-19, as well as loneliness, while chronic social isolation (i.e., “SI to SI”) was not. A possible explanation for this association is social media use. Social media use was reported to be associated with feelings of fear and threat from COVID-19 [35,36]. As those who became socially isolated during the pandemic had less contact with other people, they may have had only limited sources of information on COVID-19, such as social media, resulting in fear. We revealed that social isolation, which promotes social distancing and reduces the risk of infection, may increase the fear of COVID-19. In contrast, those who were socially isolated before and during the pandemic did not have a greater fear of COVID-19. As they had little contact with others before the pandemic, the risk of infection and fear of COVID-19 may be less.

Some limitations must be considered. First, since we used the sample collected through a web-based survey, the sample did not reflect the demographic distribution of the general population, which is the nature of a web-based survey. To adjust for potential bias in the demographic distribution of the collected sample, we used sampling weights using external, nationally representative data. However, there may be residual bias. For example, since this study was a web-based survey, those who were familiar with the internet and social networking services tended to participate in the survey, which possibly led to an underestimated prevalence of social isolation. Second, we asked the participants about their frequency of contact with “family members or relatives who are living apart” and “friends or neighbors.” However, we did not include contact with colleagues (i.e., work-related connections). This was due to the fact that the variables used to assess social isolation were based on the methods used in previous studies, particularly for the older population [18,19,20]. As the questions assessing social isolation did not include work-related connections, the prevalence of social isolation was highest in middle-aged people, and this prevalence could have been overestimated. Future studies must consider various forms of contact to assess social isolation appropriately. Third, we could not determine whether the respondents were infected with COVID-19. However, this may be a confounding factor of the association between the prevalence of social isolation and mental health outcomes. This information should be included in future studies. Fourth, since this study used a cross-sectional design, we could not clarify the direction of causality. For example, those who feared COVID-19 potentially became socially isolated since a higher risk perception of COVID-19 reduced their herding behavior [37,38]. Finally, since we asked participants about their connections with family members or relatives who were living apart, neighbors, and friends to understand their social isolation status before the pandemic, there may have been recall bias.

## 5. Conclusions

This study suggested that social isolation significantly increased during the COVID-19 pandemic in Japan. The increase was greater among older age groups and men but did not differ by SES. Social isolation during the pandemic was also associated with greater loneliness and fear of COVID-19. Effective strategies to prevent social isolation during the pandemic should be urgently investigated to maintain people’s mental health.

## Figures and Tables

**Table 1:** Weighted prevalence of social isolation before and during the COVID-19 pandemic.

Variable	Category	%	Before (January 2020)% (95% CI)	During (August 2020)% (95% CI)	Difference	
					Percentage Points (95% CI)	*p*
Total (100.0%)	All ages	100.0	21.2 (20.7–21.7)	27.9 (27.3–28.4)	6.7 (6.3–7.0)	
	15–19 years	5.7	18.0 (16.0–19.9)	21.1 (19.0–23.2)	3.1 (1.8–4.5)	<0.001
	20–29 years	12.7	20.0 (18.6–21.4)	23.5 (22.1–25.0)	3.5 (2.6–4.5)	
	30–39 years	14.2	20.6 (19.3–21.9)	26.2 (24.7–27.6)	5.6 (4.6–6.5)	
	40–49 years	18.5	24.0 (22.8–25.2)	30.9 (29.5–32.2)	6.9 (6.0–7.8)	
	50–59 years	16.4	23.5 (22.2–24.8)	31.3 (29.9–32.8)	7.8 (6.9–8.8)	
	60–69 years	16.0	19.9 (18.7–21.2)	28.0 (26.7–29.4)	8.1 (7.1–9.1)	
	70–79 years	16.5	19.7 (18.5–20.9)	27.9 (26.6–29.3)	8.2 (7.2–9.2)	
Men (49.6%)	All ages	100.0	26.8 (26.0–27.5)	34.4 (33.6–35.2)	7.6 (7.0–8.2)	
	15–19 years	5.8	23.5 (20.4–26.5)	26.3 (23.1–29.4)	2.8 (0.8–4.9)	<0.001
	20–29 years	13.1	25.2 (23.1–27.3)	30.0 (27.8–32.2)	4.8 (3.4–6.2)	
	30–39 years	14.5	27.0 (25.0–29.1)	33.1 (31.0–35.3)	6.1 (4.7–7.6)	
	40–49 years	18.8	30.4 (28.6–32.3)	38.0 (36.0–39.9)	7.6 (6.3–8.9)	
	50–59 years	16.6	28.8 (26.9–30.8)	37.7 (35.6–39.8)	8.9 (7.5–10.3)	
	60–69 years	15.8	25.8 (23.9–27.7)	34.7 (32.6–36.8)	8.9 (7.5–10.4)	
	70–79 years	15.4	23.5 (21.6–25.4)	34.0 (31.9–36.1)	10.5 (8.9–12.1)	
Women (50.4%)	All ages	100.0	15.8 (15.1–16.4)	21.4 (20.7–22.1)	5.6 (5.2–6.2)	
	15–19 years	5.6	12.3 (9.9–14.7)	15.8 (13.1–18.5)	3.5 (1.6–5.4)	<0.001
	20–29 years	12.3	14.5 (12.7–16.2)	16.7 (14.9–18.5)	2.2 (1.0–3.5)	
	30–39 years	13.9	14.1 (12.5–15.7)	19.1 (17.3–20.9)	5.0 (3.8–6.2)	
	40–49 years	18.2	17.4 (15.9–19.0)	23.6 (21.8–25.3)	6.2 (4.9–7.4)	
	50–59 years	16.3	18.2 (16.6–19.9)	25.0 (23.1–26.9)	6.8 (5.4–8.1)	
	60–69 years	16.2	14.3 (12.8–15.8)	21.6 (19.8–23.4)	7.3 (6.0–8.6)	
	70–79 years	17.6	16.4 (14.9–18.0)	22.7 (21.0–24.4)	6.3 (5.1–7.5)	

CI: Confidence interval; COVID-19: Coronavirus disease 2019.

**Table 2:** Weighted prevalence of social isolation before and during the COVID-19 pandemic by sociodemographic characteristics.

Variable	Category	%	Before (January 2020)% (95% CI)	During (August 2020)% (95% CI)	Difference	
					Percentage Points (95% CI)	*p*
Marital Status	Married	59.0	18.9 (18.3–19.6)	26.3 (25.6–27.0)	7.4 (6.9–7.9)	<0.001
	Not married	41.0	24.5 (23.7–25.4)	30.1 (29.2–31.0)	5.6 (5.0–6.2)	
Household composition	Living alone	19.8	21.5 (20.4–22.7)	26.8 (25.6–28.1)	5.3 (4.5–6.1)	0.001
	Cohabiting	80.2	21.2 (20.6–21.7)	28.1 (27.5–28.7)	6.9 (6.5–7.4)	
Education	Junior high school graduate	1.7	35.4 (30.9–39.9)	39.6 (35.0–44.2)	4.2 (1.3–7.1)	0.303
	High school graduate	28.9	24.2 (23.2–25.2)	30.9 (29.9–32.0)	6.7 (6.0–7.4)	
	Junior/vocational college graduate	22.0	19.2 (18.2–20.2)	26.2 (25.0–27.3)	7.0 (6.2–7.8)	
	University/graduate school graduate	47.4	19.8 (19.1–20.5)	26.3 (25.6–27.1)	6.5 (6.0–7.1)	
Annual household income	≤2.9 million yen	18.7	25.8 (24.5–27.0)	32.2 (30.9–33.5)	6.4 (5.5–7.3)	0.074
	3.0–4.9 million yen	21.7	19.6 (18.6–20.7)	27.0 (25.8–28.1)	7.4 (6.5–8.2)	
	5.0–6.9 million yen	15.4	18.5 (17.3–19.7)	25.0 (23.6–26.3)	6.5 (5.5–7.5)	
	7.0–9.9 million yen	14.0	17.0 (15.7–18.2)	23.3 (21.9–24.7)	6.3 (5.3–7.3)	
	≥10.0 million yen	9.4	13.8 (12.5–15.2)	19.0 (17.4–20.6)	5.2 (4.1–6.2)	
	Unknown/undisclosed	20.9	27.0 (25.8–28.2)	34.0 (32.8–35.3)	7.0 (6.2–7.9)	
Working conditions	Self-employed	9.6	20.2 (18.7–21.8)	26.5 (24.7–28.2)	6.3 (5.0–7.4)	0.112
	Permanent employment	33.2	22.1 (21.2–23.0)	28.6 (27.6–29.5)	6.5 (5.8–7.1)	
	Temporary employment	16.6	21.4 (20.2–22.7)	28.2 (26.8–29.6)	6.8 (5.8–7.7)	
	Unemployed	40.6	20.6 (19.9–21.4)	27.5 (26.6–28.3)	6.9 (6.2–7.4)	
House ownership	Yes	30.6	21.2 (20.3–22.1)	26.3 (25.4–27.3)	5.1 (4.5–5.8)	<0.001
	No	69.4	21.2 (20.6–21.8)	28.5 (27.9–29.2)	7.3 (6.8–7.8)	

CI: Confidence interval; COVID-19: Coronavirus disease 2019.

**Table 3:** Weighted prevalence of social isolation before and during the COVID-19 pandemic by environmental factors.

Variable	Category	Before (January 2020)% (95% CI)	During (August 2020)% (95% CI)	Difference	
				Percentage Points (95% CI)	*p*
Urbanization level	Highest (≥9686 people/km^2^)	21.5 (20.6–22.5)	28.7 (27.7–29.8)	7.2 (6.5–8.0)	0.070
	Second highest (6058–9685 people/km^2^)	22.0 (20.8–23.1)	28.4 (27.1–29.6)	6.4 (5.6–7.3)	
	Second lowest (≤6057 people/km^2^)	21.2 (20.1–22.2)	27.0 (25.9–28.1)	5.9 (5.1–6.6)	
	Lowest (non-DID)	19.9 (18.9–20.9)	26.7 (25.6–27.8)	6.8 (6.1–7.6)	
Prefectural COVID-19 outbreak situation	Highest (≥36.99 cases per 100,000 population)	22.5 (21.0–24.0)	29.2 (27.6–30.8)	6.7 (5.5–7.8)	0.912
	Second highest (20.33–36.98 cases per 100,000 population)	21.5 (20.3–22.7)	28.3 (27.0–29.7)	6.8 (5.9–7.7)	
	Second lowest (8.84–20.32 cases per 100,000 population)	19.9 (18.6–21.2)	26.2 (24.8–27.7)	6.3 (5.3–7.3)	
	Lowest (≤8.83 cases per 100,000 population)	21.2 (20.5–21.8)	27.8 (27.1–28.6)	6.6 (6.1–7.2)	

CI: Confidence interval; COVID-19: Coronavirus disease 2019; DID: Densely inhabited district.

**Table 4:** Weighted prevalence of the transition of social isolation before and during the COVID-19 pandemic.

Variable	Category	% (95% CI)
Total	SI to SI	19.5 (19.3–19.8)
	SI to non-SI	1.7 (1.6–1.8)
	Non-SI to SI	8.3 (8.2–8.5)
	Non-SI to non-SI	70.4 (70.2–70.7)
Men	SI to SI	24.9 (24.6–25.3)
	SI to non-SI	1.8 (1.7–2.0)
	Non-SI to SI	9.4 (9.2–9.7)
	Non-SI to non-SI	63.8 (63.4–64.2)
Women	SI to SI	14.2 (13.9–14.5)
	SI to non-SI	1.6 (1.5–1.7)
	Non-SI to SI	7.3 (7.0–7.5)
	Non-SI to non-SI	77.0 (76.6–77.4)

CI: Confidence interval; COVID-19: Coronavirus disease 2019; non-SI: Non-socially isolated; SI: Socially isolated.

**Table 5:** Weighted prevalence of social isolation before and during the COVID-19 pandemic by sociodemographic characteristics.

Variable	Psychological Distress ^a^	Suicidal Ideation ^a^	Loneliness ^b^		Fear of COVID-19 ^b^	
	OR (95% CI)	OR (95% CI)	β	*p*	β	*p*
SI to SI	0.901 (0.795–1.022)	0.687 (0.561–0.842)	0.009	0.140	−0.001	0.854
Non-SI to SI	1.013 (0.850–1.207)	1.094 (0.851–1.407)	0.020	0.001	0.028	<0.001
SI to non-SI	1.305 (0.955–1.785)	0.673 (0.380–1.194)	0.011	0.056	0.005	0.380
Non-SI to non-SI	Reference	Reference	Reference		Reference	

Β: Standardized coefficient; CI: Confidence interval; COVID-19: Coronavirus disease 2019; non-SI: Non-socially isolated; OR: Odds ratio; SI: Socially isolated. ^a^ Binary logistic regression analysis. ^b^ Multiple linear regression analysis. The model was adjusted for age, sex, marital status, household composition, education level, annual household income, working conditions, house ownership, smoking status, drinking habit, vigorous physical activity, and the presence of chronic diseases (hypertension, angina, myocardial infarction, stroke, diabetes mellitus, chronic obstructive pulmonary disease, and cancer).

## Data Availability

The datasets used and analyzed during the current study are available from the corresponding author on reasonable request.

## Supplementary Materials

The following are available online at https://www.mdpi.com/article/10.3390/ijerph18168238/s1. Figure S1: The transition of the number of newly reported COVID-19 cases from February to November 2020 in Japan; Table S1: Weighted prevalence of each social isolation item before and during the COVID-19 pandemic; Table S2: Weighted prevalence of social isolation before and during the COVID-19 pandemic by sociodemographic characteristics, by sex.
