# Supplementary material for: Increase in Social Isolation during the COVID-19 Pandemic and Its Association with Mental Health: Findings from the JACSIS 2020 Study
Source: Int J Environ Res Public Health. 2021 Aug 4;18(16):8238. doi: 10.3390/ijerph18168238 (PMC8394951; doi:10.3390/ijerph18168238)
Supplement: Supplementary file 1 [file ijerph-18-08238-s001.zip › Supplementary material_Figure S1_IJERPH_2.pptx]

## Slide 1
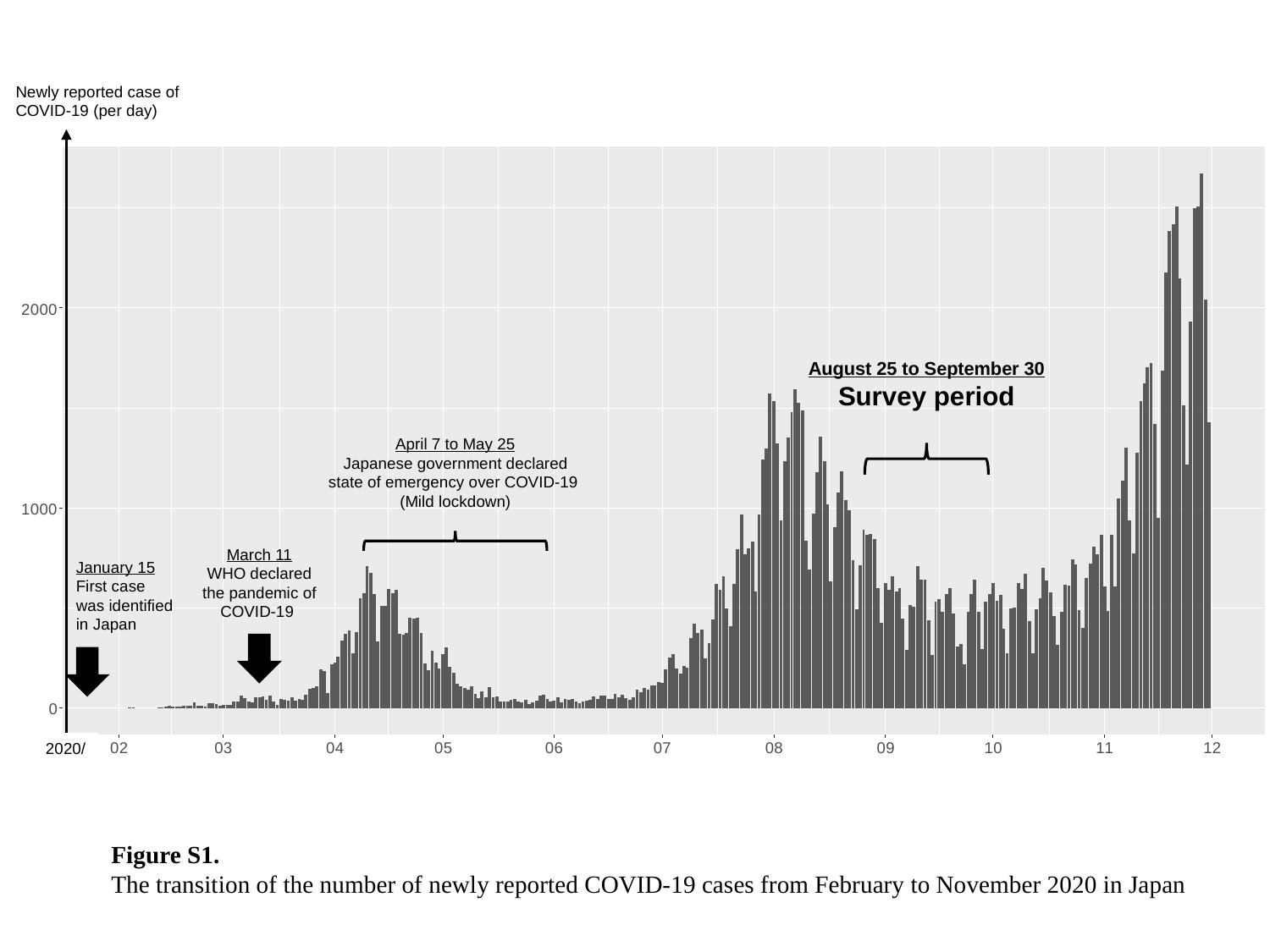

Newly reported case of COVID-19 (per day)
August 25 to September 30
Survey period
April 7 to May 25
Japanese government declared state of emergency over COVID-19
(Mild lockdown)
March 11
WHO declared the pandemic of COVID-19
January 15
First case was identified in Japan
2020/
Figure S1.
The transition of the number of newly reported COVID-19 cases from February to November 2020 in Japan
