# Supplementary material for: Increase in Social Isolation during the COVID-19 Pandemic and Its Association with Mental Health: Findings from the JACSIS 2020 Study
Source: Int J Environ Res Public Health. 2021 Aug 4;18(16):8238. doi: 10.3390/ijerph18168238 (PMC8394951; doi:10.3390/ijerph18168238)
Supplement: Supplementary file 1 [file ijerph-18-08238-s001.zip › Supplementary_material_Table_S1-S2_IJERPH_2.pdf]

Table S1. Weighted prevalence of each social isolation item before and during the COVID-19 pandemic

		Before (January 2020)							During (August 2020)						
		**Almost every day**	**4–5 times a week**	**2–3 times a week**	**Once a week**	**2–3 times a month**	**Once a month**	**Rarely**	**Almost every day**	**4–5 times a week**	**2–3 times a week**	**Once a week**	**2–3 times a month**	**Once a month**	**Rarely**
(1) How often did you see your family members or relatives who are living apart?															
Total		1.5	1.3	3.7	7.9	12.3	31.1	42.2	1.2	1.1	3.1	6.6	9.3	22.3	56.4
Men	All ages	1.4	1.1	3.0	7.0	10.8	29.6	47.1	1.0	1.0	2.6	6.1	8.6	20.9	59.8
	15–19 years	1.0	2.0	4.8	7.0	8.9	20.6	55.8	1.6	2.0	3.5	8.4	8.0	14.0	62.3
	20–29 years	0.8	0.7	2.5	7.2	11.3	26.6	50.9	0.8	1.2	3.9	6.5	9.5	17.0	61.1
	30–39 years	0.8	0.8	2.8	7.8	11.3	29.8	29.8	0.7	0.9	2.4	6.6	11.1	21.4	56.8
	40–49 years	1.6	1.3	2.6	7.6	9.6	27.2	50.0	1.0	0.7	2.3	6.6	7.9	19.8	61.8
	50–59 years	1.9	0.9	2.5	6.1	10.8	30.2	47.6	1.0	1.0	2.4	4.4	8.1	21.7	61.5
	60–69 years	1.7	1.4	3.6	6.5	10.9	34.3	41.6	1.4	1.2	2.4	5.3	7.4	24.4	57.9
	70–79 years	1.6	1.1	3.0	7.0	11.9	32.7	42.5	1.0	0.8	2.0	6.4	8.6	23.0	58.2
Women	All ages	1.7	1.4	4.4	8.7	13.9	32.6	37.4	1.4	1.2	3.6	7.1	9.9	23.7	53.1
	15–19 years	1.7	0.8	2.7	5.5	13.3	26.2	49.9	1.5	0.8	2.9	5.5	8.7	20.7	59.9
	20–29 years	0.8	1.0	3.3	7.6	13.0	32.8	41.5	1.3	0.9	2.6	7.1	12.2	24.5	51.4
	30–39 years	1.3	2.1	4.8	9.0	15.8	33.8	33.2	1.1	1.4	5.0	8.1	11.1	25.4	47.9
	40–49 years	2.1	1.2	4.0	8.9	14.5	31.0	38.1	1.8	1.2	2.9	7.9	10.0	23.7	52.6
	50–59 years	2.0	1.2	4.0	8.6	13.5	32.9	37.9	1.7	1.1	3.7	6.7	9.9	22.6	54.2
	60–69 years	2.1	1.7	5.9	10.8	14.2	33.3	31.9	1.7	1.5	3.8	7.4	9.5	23.8	52.3
	70–79 years	1.4	1.5	4.8	8.2	12.4	34.0	37.6	0.7	1.1	3.5	6.1	8.2	23.6	56.8
(2) How often did you make contact with your family members or relatives who are living apart by email or text message?															
Total		15.7	6.2	10.6	11.4	12.3	11.2	32.5	12.9	5.9	10.6	11.0	11.8	12.5	35.2
Men	All ages	11.5	4.9	8.7	11.0	12.5	12.3	39.1	9.4	4.4	8.9	10.0	11.6	13.3	42.3
	15–19 years	11.0	5.9	8.4	10.4	10.2	9.7	44.4	9.4	5.9	10.8	12.3	7.8	9.0	44.9
	20–29 years	8.9	5.3	9.0	13.7	13.4	13.7	36.0	7.7	4.7	10.1	11.8	12.5	13.8	39.5
	30–39 years	10.6	3.8	8.2	12.8	17.1	13.5	34.0	8.5	3.9	9.0	12.6	14.9	13.9	37.3
	40–49 years	11.3	3.9	7.5	10.9	12.3	13.3	40.8	9.7	4.0	7.9	9.2	11.4	13.6	44.2
	50–59 years	13.5	4.8	7.7	9.0	11.1	11.4	42.5	10.7	3.8	8.3	8.4	9.5	13.7	45.7
	60–69 years	13.0	5.9	11.0	8.9	11.2	11.7	38.3	10.4	5.6	9.6	8.3	10.9	13.4	41.9
	70–79 years	11.3	5.2	9.3	11.5	11.6	11.2	39.9	9.0	4.2	8.5	10.0	12.3	13.4	42.6

Women	All ages	19.9	7.6	12.5	11.8	12.0	10.1	26.0	16.4	7.4	12.3	11.9	12.0	11.7	28.3
	15–19 years	22.2	6.3	8.8	9.8	10.5	9.0	33.4	18.6	7.3	9.4	10.2	8.8	9.9	35.8
	20–29 years	22.7	7.5	11.7	11.6	11.9	10.6	24.0	19.7	8.8	11.8	11.4	11.6	10.7	26.0
	30–39 years	21.1	8.4	12.8	14.1	13.6	10.9	19.1	18.5	7.7	13.8	13.3	13.3	11.5	21.9
	40–49 years	17.5	6.1	13.0	12.0	13.8	11.8	25.7	14.9	6.3	12.0	12.4	13.5	12.3	28.6
	50–59 years	21.1	7.0	11.1	10.3	11.3	11.0	28.2	17.3	6.9	11.6	10.2	10.5	12.8	30.6
	60–69 years	20.2	8.3	14.0	11.4	10.9	9.3	25.9	15.8	7.5	14.9	11.0	11.4	12.8	26.5
	70–79 years	17.3	8.7	13.6	12.3	11.2	7.8	29.1	12.9	7.8	11.2	13.4	12.9	10.3	31.5
(3) How often did you make contact with your family members or relatives who are living apart by voice call?															
Total		4.9	3.6	8.1	11.9	14.0	18.8	38.5	5.0	3.5	7.8	11.2	12.6	16.6	43.3
Men	All ages	3.6	2.8	6.6	11.0	13.7	18.8	43.4	3.7	2.8	6.5	10.0	12.1	16.5	48.5
	15–19 years	4.6	3.4	7.8	12.3	11.0	10.6	50.3	5.6	3.3	7.4	10.6	10.6	8.4	54.1
	20–29 years	2.5	2.4	6.7	12.3	12.4	17.1	46.6	2.8	2.7	7.5	10.5	12.0	14.5	49.9
	30–39 years	3.4	2.6	6.4	11.2	15.2	18.3	43.0	3.7	2.5	7.1	10.5	12.2	15.9	48.2
	40–49 years	3.3	3.0	6.1	11.4	12.5	18.9	44.9	3.7	2.6	6.0	10.8	10.8	15.9	50.2
	50–59 years	4.4	2.8	6.0	10.4	12.6	18.0	45.8	4.1	2.8	5.4	8.8	11.3	16.5	51.1
	60–69 years	3.5	3.2	7.2	9.2	13.5	22.0	41.6	3.6	3.5	6.6	8.8	11.4	18.9	47.3
	70–79 years	3.9	2.8	7.2	11.5	17.2	21.3	36.1	3.1	2.4	6.7	10.7	15.6	19.9	41.5
Women	All ages	6.2	4.4	9.6	12.8	14.3	18.9	33.7	6.4	4.2	9.1	12.3	13.1	16.8	38.1
	15–19 years	8.3	3.5	8.5	9.1	11.2	13.1	46.3	8.4	3.2	7.8	10.3	8.7	10.8	50.8
	20–29 years	6.7	4.6	8.3	10.7	13.2	17.2	39.2	6.5	4.3	8.8	11.4	10.8	16.0	42.3
	30–39 years	6.7	5.2	9.2	13.1	13.8	19.4	32.6	7.2	4.3	9.4	12.3	13.5	16.0	37.3
	40–49 years	5.6	3.7	8.6	12.9	14.3	18.9	36.1	5.1	4.2	7.7	12.9	12.7	15.9	41.5
	50–59 years	7.3	4.2	8.9	12.6	13.4	19.2	34.5	7.0	4.5	8.9	10.8	12.2	16.5	40.1
	60–69 years	5.6	4.8	11.1	13.8	14.3	21.4	28.9	6.4	4.1	10.2	13.3	14.2	19.1	32.8
	70–79 years	5.2	4.6	11.2	14.6	17.3	18.9	28.2	5.7	4.3	10.1	13.3	16.2	18.9	31.4
(4) How often did you make contact with your family members or relatives who are living apart by video call?															
Total		1.4	1.3	2.7	4.8	5.1	7.8	76.9	1.8	1.4	3.0	5.0	5.3	7.7	75.7
Men	All ages	1.1	1.0	2.4	4.6	5.4	7.5	78.0	1.2	1.2	2.6	4.9	5.3	7.3	77.5
	15–19 years	3.3	1.8	6.0	7.4	7.2	6.8	67.5	2.6	1.8	6.0	8.0	7.6	7.0	67.0
	20–29 years	1.0	1.8	3.8	6.5	8.3	10.0	68.5	1.5	2.1	4.2	7.7	7.6	9.3	67.5
	30–39 years	1.3	1.4	3.0	6.7	7.6	8.5	71.5	1.4	1.9	3.4	7.3	7.6	9.2	69.2
	40–49 years	0.8	0.9	2.1	3.8	4.6	6.1	81.7	1.1	0.8	2.1	4.4	4.3	6.9	80.4
	50–59 years	1.0	0.6	1.0	2.8	3.1	5.3	86.2	1.0	0.5	1.1	2.7	3.4	4.9	86.3
	60–69 years	0.8	0.8	2.1	3.3	4.3	8.3	80.5	1.1	0.9	2.5	3.1	4.0	7.5	81.1
	70–79 years	0.9	0.6	1.6	4.4	4.9	7.6	80.1	0.9	0.9	1.9	3.8	4.7	6.9	80.9

Women	All ages	1.6	1.6	3.0	5.1	4.7	8.1	75.8	2.4	1.7	3.4	5.0	5.4	8.2	73.9
	15–19 years	3.5	2.5	4.5	5.5	7.4	8.5	68.1	4.9	3.1	4.2	6.3	7.6	8.0	66.0
	20–29 years	2.5	2.3	3.7	7.2	7.0	9.5	67.8	3.6	3.2	5.1	6.7	7.1	10.0	64.2
	30–39 years	2.1	3.0	4.5	7.3	7.0	10.1	65.9	3.6	2.1	5.1	7.2	8.2	12.3	61.7
	40–49 years	1.5	1.2	2.3	3.8	3.3	6.7	81.3	1.8	1.1	2.7	3.8	4.3	6.4	79.9
	50–59 years	1.1	1.1	1.7	3.4	2.5	5.5	84.5	1.5	1.2	2.2	3.1	3.1	5.7	83.2
	60–69 years	1.3	1.2	3.4	5.3	5.2	8.5	75.1	1.7	2.0	3.3	5.7	4.9	8.7	73.7
	70–79 years	1.1	1.2	2.5	4.2	3.6	8.7	78.6	1.9	0.8	2.6	4.2	4.9	7.3	78.3
(5) How often did you see your friends or neighbors?															
Total		2.8	2.8	5.6	8.9	16.5	28.0	35.3	1.4	1.4	3.4	6.0	9.5	18.1	60.1
Men	All ages	2.8	2.6	5.2	9.0	15.3	25.5	39.7	1.6	1.5	3.4	6.5	9.1	16.4	61.6
	15–19 years	16.1	10.2	9.8	8.6	12.7	12.4	30.2	9.8	8.0	9.3	9.7	11.4	12.4	39.4
	20–29 years	4.0	5.4	7.6	12.2	17.0	19.9	34.0	1.8	2.4	6.6	10.7	12.5	17.6	48.5
	30–39 years	0.9	1.5	4.2	10.0	15.8	27.4	40.3	0.5	0.9	3.1	7.4	9.1	16.6	62.3
	40–49 years	1.8	1.4	4.3	8.9	13.0	25.0	45.6	1.1	0.8	2.6	6.2	7.6	15.5	66.3
	50–59 years	2.3	1.4	4.4	7.8	14.0	27.4	42.6	1.3	1.0	2.5	4.3	7.4	15.9	67.6
	60–69 years	1.7	1.9	4.0	7.2	15.8	29.5	40.0	0.9	0.8	1.8	4.5	7.8	18.0	66.2
	70–79 years	1.6	1.7	5.4	8.8	17.8	27.7	37.0	0.8	0.9	2.7	5.3	10.0	16.8	63.5
Women	All ages	2.8	3.1	6.0	8.9	17.8	30.5	30.9	1.3	1.3	3.4	5.6	9.9	19.8	58.6
	15–19 years	20.0	12.3	9.1	9.4	14.4	14.5	20.3	12.2	7.1	8.8	9.4	16.4	13.0	33.1
	20–29 years	5.1	5.4	7.4	10.9	23.0	22.8	25.2	1.3	1.8	4.7	9.3	16.2	24.0	42.7
	30–39 years	1.0	2.0	5.4	8.2	19.5	32.8	31.2	0.5	0.9	3.1	5.4	10.8	20.7	58.5
	40–49 years	1.2	1.3	3.9	8.1	16.6	32.9	36.1	0.4	0.9	2.3	4.9	7.5	20.0	64.0
	50–59 years	1.4	1.8	3.9	6.5	14.9	35.8	35.6	0.7	0.7	1.9	3.2	6.7	18.0	68.8
	60–69 years	1.2	2.1	6.4	9.3	15.9	34.5	30.7	0.7	0.8	2.6	4.6	7.8	21.3	62.3
	70–79 years	1.7	3.3	8.4	10.7	19.6	28.0	28.4	0.6	1.1	4.4	6.0	9.9	18.5	59.5
(6) How often did you make contact with your friends or neighbors by email or text message?															
Total		19.5	7.6	12.3	11.1	11.7	10.2	27.7	16.1	6.5	11.1	10.4	11.7	12.0	32.2
Men	All ages	15.8	6.7	11.0	11.0	11.6	10.8	32.9	13.3	5.5	9.8	9.8	11.3	12.2	38.0
	15–19 years	27.0	9.3	13.1	8.6	7.8	6.4	27.8	25.4	8.8	11.0	12.2	6.8	6.8	28.8
	20–29 years	20.2	9.1	10.6	12.4	12.7	9.0	26.0	17.2	7.8	10.5	11.3	11.9	9.9	31.4
	30–39 years	14.7	5.7	11.2	14.0	13.4	12.2	28.7	12.1	5.1	10.1	11.5	13.3	13.2	34.6
	40–49 years	16.1	7.7	11.3	10.2	11.6	11.3	31.9	13.4	6.1	10.3	9.7	10.8	11.7	38.0
	50–59 years	17.2	6.7	10.5	10.8	11.0	9.7	34.1	14.5	5.2	10.2	8.7	10.5	11.7	39.3
	60–69 years	12.8	6.2	11.3	10.0	11.3	11.9	36.5	10.1	4.8	9.1	8.7	11.5	14.4	41.3
	70–79 years	10.1	4.2	10.3	10.4	11.7	12.3	41.0	8.7	3.1	8.4	8.6	12.1	13.9	45.3

Women	All ages	23.1	8.4	13.5	11.1	11.8	9.6	22.5	18.8	7.5	12.3	11.0	12.1	11.8	26.5
	15–19 years	46.9	7.4	10.6	7.8	7.4	3.2	16.6	41.4	9.1	9.8	8.0	8.0	4.8	19.0
	20–29 years	38.7	8.9	11.9	8.5	9.1	7.2	15.8	32.8	10.3	11.9	9.0	10.2	7.6	18.2
	30–39 years	22.1	9.2	14.7	14.0	13.8	10.1	16.1	18.7	7.4	13.6	12.0	14.9	11.7	21.7
	40–49 years	22.3	8.7	14.5	11.2	12.3	11.0	19.9	18.3	7.6	12.9	10.9	12.2	11.9	26.3
	50–59 years	21.9	8.2	13.1	10.8	12.5	10.6	22.8	17.4	7.1	11.3	10.5	11.8	14.6	27.3
	60–69 years	15.7	8.2	13.3	13.0	13.0	11.4	25.4	11.5	6.9	13.5	12.1	12.7	15.1	28.2
	70–79 years	14.1	7.8	14.2	10.1	11.3	8.9	33.6	10.4	5.9	11.5	11.9	12.4	11.4	36.5
(7) How often did you make contact with your friends or neighbors by voice call?															
Total		4.5	3.3	7.1	10.4	12.8	17.7	44.3	4.6	2.9	6.4	9.2	11.0	15.2	50.6
Men	All ages	3.9	2.8	7.1	10.3	12.8	17.4	45.7	4.1	2.7	6.1	9.4	10.9	14.8	52.0
	15–19 years	7.6	4.4	11.1	11.9	11.5	10.8	42.8	7.8	5.9	7.6	12.7	11.9	8.2	46.0
	20–29 years	3.9	3.6	7.5	11.4	13.6	16.6	43.5	4.8	3.6	7.6	11.0	11.8	14.2	47.1
	30–39 years	3.4	2.7	6.3	11.4	13.3	16.8	46.1	3.5	2.5	6.5	10.7	10.8	15.6	50.4
	40–49 years	4.2	2.5	7.1	10.6	11.1	15.6	48.9	4.5	2.5	5.4	10.0	8.8	13.0	55.8
	50–59 years	4.4	2.9	6.2	8.7	12.3	15.6	49.9	4.6	2.5	4.8	7.9	10.2	14.1	56.0
	60–69 years	2.8	2.7	7.2	8.2	11.9	20.4	46.9	3.1	2.4	6.3	7.1	11.0	16.3	54.0
	70–79 years	3.3	2.2	6.9	10.9	15.5	22.5	38.7	2.5	1.8	5.9	8.7	13.4	18.5	49.1
Women	All ages	5.0	3.7	7.0	10.5	12.8	18.0	43.0	5.1	3.2	6.8	9.1	11.0	15.7	49.2
	15–19 years	10.6	5.3	8.8	9.8	13.4	14.5	37.5	11.2	5.6	9.0	10.2	10.2	11.0	42.8
	20–29 years	7.5	4.8	8.0	11.0	12.5	15.3	40.9	8.0	4.6	8.0	10.0	11.3	15.0	43.0
	30–39 years	4.7	3.6	5.8	9.9	10.7	17.3	48.0	4.6	2.6					
